# Application of Clinico-Radiologic-Pathologic Diagnosis of Diffuse Parenchymal Lung Diseases in Children in China

**DOI:** 10.1371/journal.pone.0116930

**Published:** 2015-01-08

**Authors:** Dan Xu, Zhimin Chen, Huizhong Chen, Rongyan Huang, Shunying Zhao, Xiuyun Liu, Chunju Zhou, Yun Peng, Xinyu Yuan, Jizhen Zou, Hailing Zhang, Deyu Zhao, Enmei Liu, Yuejie Zheng, Lili Zhong, Min Lu, Jirong Lu, Guangmin Nong

**Affiliations:** 1 Department of Pulmonology, Children’s Hospital, Zhejiang University School of Medicine, Hangzhou, China; 2 Department of Respiratory Diseases, Children’s Hospital, Affiliated to the Capital Institute of Pediatrics, Beijing, China; 3 Department of Pediatric Internal Medicine, Beijing Children’s Hospital Affiliated with Capital Medical University, Beijing, China; 4 Department of Pathology, Beijing Children’s Hospital Affiliated with Capital Medical University, Beijing, China; 5 Department of Radiology, Beijing Children’s Hospital Affiliated with Capital Medical University, Beijing, China; 6 Department of Radiology, Children’s Hospital, Affiliated to the Capital Institute of Pediatrics, Beijing, China; 7 Department of Pathology, C Children’s Hospital, Affiliated to the Capital Institute of Pediatrics, Beijing, China; 8 Department of Respiratory Diseases, Yuying Children’s Hospital Affiliated to Wenzhou Medical College, Wenzhou, China; 9 Department of Respiratory Diseases, Nanjing Children’s Hospital, Nanjing Medical University, Nanjing, China; 10 Respiratory Center, Children’s Hospital Affiliated with Chongqing Medical University, Chongqing, China; 11 Department of Respiratory Diseases, Shenzhen Children’s Hospital, Shenzhen, China; 12 Department of Pediatrics, the First Affiliated Hospital of Hunan Normal University, Changsha, China; 13 Department of Pulmonology, Shanghai Children’s Hospital Affiliated to Jiaotong University, Shanghai, China; 14 Department of Pediatrics, the First Hospital of Jilin University, Changchun, China; 15 Department of Pediatrics, the First Affiliated Hospital, Guangxi Medical University, Nanning, China; Fondazione IRCCS Ca’ Granda Ospedale Maggiore Policlinico, Università degli Studi di Milano, ITALY

## Abstract

Diffuse parenchymal lung diseases in children (chDPLD) or interstitial lung diseases in children (chILD) represent a heterogeneous group of respiratory disorders that are mostly chronic and associated with high morbidity and mortality. However, the incidence of chDPLD is so low that most pediatricians lack sufficient knowledge of chDPLD, especially in China. Based on the clinico- radiologic- pathologic (CRP) diagnosis, we tried to describe (1) the characteristics of chDPLD and (2) the ratio of each constituent of chDPLD in China. Data were evaluated, including clinical, radiographic, and pathologic results from lung biopsies. We collected 25 cases of chDPLD, 18 boys and 7 girls with a median age of 6.0 years, from 16 hospitals in China. The most common manifestations included cough (n = 24), dyspnea (n = 21), and fever (n = 4). There were three cases of exposure-related interstitial lung disease (ILD), three cases of systemic disease-associated ILD, nineteen cases of alveolar structure disorder-associated ILD, and no cases of ILD specific to infancy. Non-specific interstitial pneumonia (n = 9) was the two largest groups. Conclusion: Non-specific interstitial pneumonia is the main categories of chDPLD in China. Lung biopsy is always a crucial step in the final diagnosis. However, clinical and imaging studies should be carefully evaluated for their value in indicating a specific chDPLD.

## Introduction

Diffuse parenchymal lung diseases in children (chDPLD) or interstitial lung diseases in children (chILD) represent a heterogeneous group of respiratory disorders that are mostly chronic and associated with high morbidity and mortality [1, 2]. They are characterized by impaired gas exchange and diffuse infiltrates on chest radiology. Clinically, children with these disorders may present with cough and dyspnea with evidence of a restrictive ventilatory defect [1].

As is the case for all diseases, the first step is to obtain a diagnosis. However, the diagnosis of chDPLD is highly challenging. The incidence of chDPLD is so low that most pediatricians lack sufficient knowledge of chDPLD. Furthermore, the clinico-radiologic-pathologic (CRP) diagnosis requires pathologic evidence, and pathologic standards in children are very difficult to obtain.

The second challenge is the classification of chDPLD, because of not only the broad spectrum of this disease but also no classification guideline for this disease in children. The American Thoracic Society/European Respiratory Society has created classification guidelines for chDPLD in adults [3]. Using this classification, patients with DPLD are separated into several categories, including (1) DPLD of known cause, (2) idiopathic interstitial pneumonias, (3) granulomatous DPLD, and (4) other forms of DPLD. However, adult classifications are not fully suitable for cases of chDPLD for the following reasons: (1) compared to adult cases, pediatric cases comprise a broader spectrum of disorders with a more variable clinical course, (2) pediatric histologic patterns often do not resemble those of adults, and (3) some forms are only observed in children [1].

Due to the above difficulties, a general description of chDPLD in China is still lacking. Therefore, we conducted a multicenter clinical investigation, based on the CRP diagnosis, aiming to describe (1) the characteristics of chDPLD, and (2) the ratio of each constituent of chDPLD in China.

## Materials and Methods

Between January 2009 and December 2011, we collected cases of chDPLD, from sixteen children’s hospitals or pediatric departments of general hospitals in China. Including criteria were: 1) signs and symptoms of respiratory diseases, including cough, wheezing, dyspnea, et al; 2) patients with high-resolution chest computerized tomography (CT) suggesting diffuse parenchymal lung diseases. Patients subjected to high-resolution CT because of either chest X-ray also suggesting diffuse parenchymal lung diseases, or disagreement between subtle changes on chest X-ray and severe symptoms; 3) patients having had lung biopsies.

Case information, including clinical, radiographic, and pathologic results from all diagnostic lung biopsies, was collected by a standardized data system after obtaining written approval from the patients’ parents or guardians. The patients’ information was anonymized and de-identified prior to analysis. The study was approved by the Ethics Committee of Children’s Hospital, Zhejiang University School of Medicine.

Members of the Pediatric Diffuse Parenchymal Lung Disease/Pediatric Interstitial Lung Disease Cooperative Group, the Subspecialty Group of Respiratory Diseases, the Society of Pediatrics, and the Chinese Medical Association, including pediatricians, radiologists and pathologists participated in the discussion to reach a final CRP diagnosis for each case according to the Multidisciplinary Consensus proposed by American Thoracic Society/European Respiratory Society [1, 3]. In every meeting, doctors providing the case would get suggestions from other specialists on further investigation if the diagnosis was still uncertain. In the next meeting, this case would be discussed again. Those with no agreement on diagnosis after three rounds of discussions within the group were excluded.

## Results

### 1. General information

**Figure 1:**
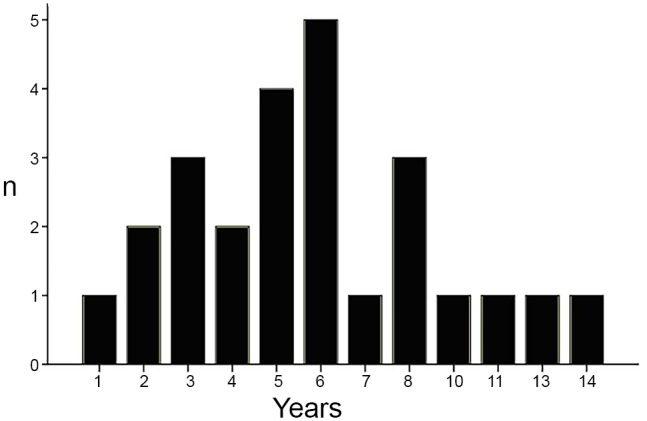
The age distribution of patients with diffuse parenchymal lung diseases (chDPLDs). The numbers of patients younger than 3 years, 3 to 7 years, and older than 7 years were 3 (12%), 14 (56%), and 8 (32%), respectively.

Twenty-seven patients were enrolled in the study. Two were excluded because of no agreement on diagnosis. In the left 25 cases, there were 18 boys and 7 girls, ranging in age from 1 year 5 months to 14 years, with a median age of 6.0 years. The numbers of patients younger than 3 years, 3 to 7 years, and older than 7 years were 3 (12%), 14 (56%), and 8 (32%), respectively (Fig. 1).

Our classification of chDPLD was mainly based on the study by Deutsch et al [3, 4] (Table 1). In our study, the most common chDPLD was non-specific interstitial pneumonia (NSIP) (n = 9), followed by acute interstitial pneumonia (AIP) (n = 4), pulmonary alveolar proteinosis (PAP) (n = 2) and extrinsic allergic alveolitis (EAA) (n = 2). Other types of chDPLD were rare with only one case of each disease.

**Table 1:** Classification of diffuse parenchymal lung diseases in children (chDPLD).

Exposure-related interstitial lung disease (ILD)
Extrinsic allergic alveolitis (EAA) (n = 2)
Lipoid pneumonia (n = 1)
Systemic disease-associated ILD
Pulmonary alveolar microlithiasis (PAM) (n = 1)
ANCA-associated vasculitis-related chDPLD (n = 1)
SLE-related chDPLD (n = 1)
Alveolar structure disorder-associated ILD
Pulmonary alveolar proteinosis (PAP) (n = 2)
Post-infectious processes (n = 1)
AIDS-related chDPLD (n = 1)
Non-specific interstitial pneumonia (NSIP) (n = 9)
Lymphocytic interstitial pneumonia (LIP) (n = 1)
Bronchiolitis obliterans organizing pneumonia (BOOP) (n = 1)
Acute interstitial pneumonia (AIP) (n = 4)
ILD specific to infancy
None

ANCA: Antineutrophil cytoplasmic antibodies;

SLE: Systemic lupus erythematosus;

AIDS: Acquired immune deficiency syndrome.

General information and basic clinico-radiologic-pathologic data from all patients are listed in Table 2. In the group of patients younger than 3 years, all were diagnosed with AIP. Interestingly, in the group of patients older than 7 years, 5 out of 8 were diagnosed with NSIP.

**Table 2:** The clinico-radiologic-pathologic data of children with diffuse parenchymal lung diseases (chDPLD).

**No.**	**Sex**	**Age**	**Clinical course (m)**	**PaO2 (kPa)**	**Lung function**	**CT scan**	**Pathology**	**CRP diagnosis**
1	F	5Y9M	9	<8		GGO, paving-stone sign	PAP	PAP
2	M	4Y	15	<8	RVD	GGO, paving-stone sign	PAP	PAP
3	M	8Y2M	9	Normal	RVD	GGO, cystic air space	Interstitial chronic inflammation	Post-infectious chDPLD
4	M	3Y2M	20 days	8–10.67		GGO	EAA	EAA
5	M	13Y1M	12	<8	RVD	GGO, reticulation	EAA	EAA
6	M	6Y	12	Normal	RVD	GGO, cystic air space	Lipoid pneumonia	Lipoid pneumonia
7	M	6Y	60	8–10.67	Normal	GGO, diffuse small nodules	PAM	PAM
8	M	5Y11M	2	8–10.67		GGO	Capillary proliferation	ANCA-associated vasculitis-related chDPLD
9	F	3Y6M	7 days			GGO, consolidation	AIP	SLE-related chDPLD
10	M	5Y9M	12			GGO	LIP	AIDS-related chDPLD
11	M	8Y2M	1	8–10.67		GGO, traction bronchiectasis	NSIP	NSIP (cellular)
12	F	4Y	2	8–10.67		GGO	NSIP	NSIP (cellular)
13	M	7Y	3	8–10.67		GGO, reticulation	NSIP	NSIP (mixed)
14	M	6Y	36	8–10.67	MVD	GGO, cystic air space	NSIP	NSIP (cellular)
15	M	6Y	5	8–10.67		GGO, reticulation	NSIP	NSIP (cellular)
16	M	10Y	84	Normal	RVD	GGO	NSIP	NSIP (mixed)
17	F	11Y	2	<8	MVD	GGO, reticulation	NSIP	NSIP (cellular)
18	M	8Y	24	8–10.67	MVD	GGO, reticulation	NSIP	NSIP (mixed)
19	F	3Y	1		Normal	GGO	NSIP	NSIP (mixed)
20	M	6Y	48	8–10.67	MVD	GGO, cystic air space	LIP	LIP
21	M	5Y	1	Normal	Normal	GGO	BOOP	BOOP
22	M	1Y5M	1			GGO, consolidation	AIP	AIP
23	F	2Y10M	1			GGO, consolidation	AIP	AIP
24	F	2Y	20 days	<8	RVD	GGO, consolidation	AIP	AIP
25	M	14Y7M	1		RVD	GGO, consolidation	AIP	AIP

F, female; M, male; m, months; RVD, restrictive ventilatory dysfunction; MVD, mixed ventilatory dysfunction; GGO, ground glass opacity; PAP, pulmonary alveolar proteinosis; EAA, extrinsic allergic alveolitis; PAM, pulmonary alveolar microlithiasis; AIP, acute interstitial pneumonia; LIP, lymphocytic interstitial pneumonia; NSIP, non-specific interstitial pneumonia; BOOP, bronchiolitis obliterans organizing pneumonia; AIP, acute interstitial pneumonia.

### 2. Clinical and laboratory findings

The duration before diagnosis ranged from 7 days to 7 years, with a median of 3 years. Nineteen cases (76%) were diagnosed within the first year of the disease course.

The main complaints included cough (n = 24), dyspnea (n = 21), and fever (n = 4). The only patient without cough had dyspnea and a final diagnosis of AIP. The four patients without dyspnea had diagnoses of BOOP, PAM, post-infectious chDPLD, and lipoid pneumonia. The four patients with fever had diagnoses of EAA, NSIP, post-infectious chDPLD, and ANCA-associated vasculitis-related chDPLD. Other symptoms included arthralgia (1 patient, NSIP), wheezing (1 patient, AIP), and short stature (1 patient, LIP).

Upon physical examination, retractions (n = 13) and nail clubbing (n = 8) were the most common signs. The eight patients with nail clubbing had diagnoses of NSIP (n = 4), PAP (n = 2), lipoid pneumonia (n = 1) and LIP (n = 1). On auscultation, 16 patients had no rales, seven had rales, two had wheezes (AIP and EAA), and two had decreased breath sounds (NSIP and EAA). The seven patients with rales had diagnoses of ANCA-associated vasculitis-related chDPLD (n = 1), SLE-related chDPLD (n = 1), EAA (n = 1), NSIP (n = 3), and AIP (n = 1). Only two patients (ANCA-associated vasculitis-related chDPLD and NSIP) had crackles typical of DPLD.

Nineteen patients had records of blood gas analysis. Four patients (post-infectious chDPLD, lipoid pneumonia, NSIP, and BOOP) appeared normal in blood gas analysis. Five patients had hypoxemia (PaO2 <8 kPa) (2 cases of PAP, and 1 each of EAA, NSIP, and AIP). The PaO2 of the remaining 10 patients was between 8 and 10.67 kPa.

In pathogen detection, one patient with AIP was positive for *Chlamydia pneumoniae*-IgM, and one patient with NSIP was positive for respiratory syncytial virus antigen. The patient with AIDS and chDPLD was positive for HIV antibody. In post-infectious patients with chDPLD, *Aspergillus fumigatus* IgG was positive, and IgM was negative. In the remaining patients, detection of pathogens, including *Mycoplasma pneumonia*, *Chlamydia trachomatis*, *Mycobacterium tuberculosis*, cytomegalovirus, Epstein-Barr virus, enteric cytopathogenic human orphan virus, coxsackievirus, respiratory syncytial virus, adenovirus, influenza virus, parainfluenza virus, and fungus, was negative.

C-ANCAs were present in the patient with ANCA-associated vasculitis-related chDPLD. In the patient with SLE-related chDPLD, anti-double-stranded DNA antibodies, anti-single-stranded DNA antibodies, and anti-SS-A/RO52KD+ were all positive. In other patients, autoimmune antibodies were all negative.

### 3. Imaging results

**Figure 2:**
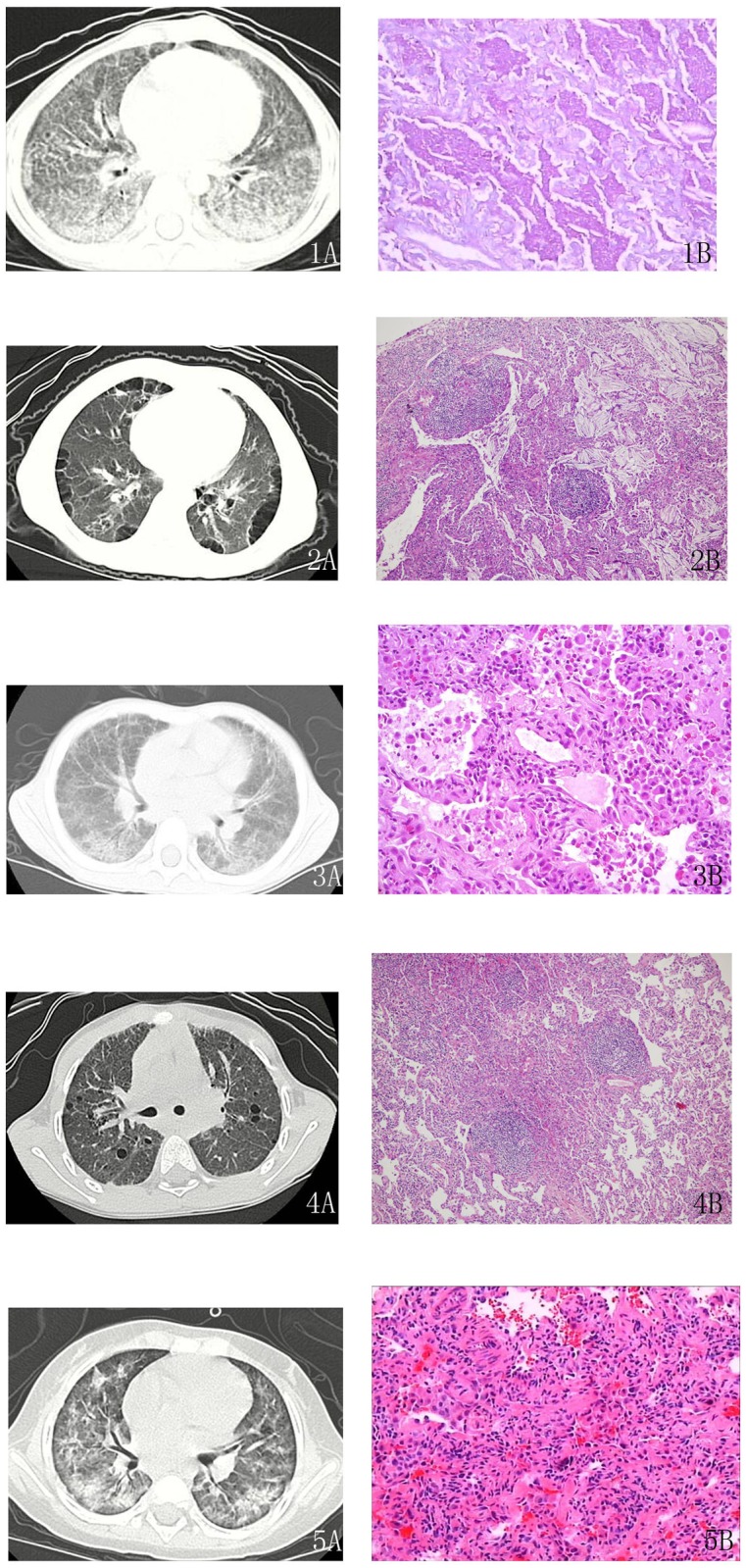
Chest tomography (CT) images and pathology results of several cases. 1A: The CT shows a paving stone sign and air bronchograms in patients with pulmonary alveolar proteinosis (case 2 in Table 2). 1B: Under light microscopy, the case of pulmonary alveolar proteinosis (case 2) shows evidence of periodic acid-Schiff-positive material filling the alveoli. Interstitial cell infiltrates, including lymphocytes and plasma cells with type II cell hyperplasia, are found. 2A: Multiple thin-walled cysts are seen in the subpleural region in the CT image of lipoid pneumonia (case 6). 2B: Light microscopy of lipoid pneumonia (case 6) shows a large amount of cholesterol crystallization in the alveolar spaces with lymphoid follicles in the alveolar septa. 3A: The CT image of non-specific interstitial pneumonia (case 15) shows reticulation on the background of ground glass opacity and interlobular septal thickening. 3B: A typical pathology picture of cellular non-specific interstitial pneumonia (case 15). The lungs are uniformly involved. Interstitial chronic inflammation consists of lymphocytes and plasma cells. 4A: On the background of ground glass opacity, thin-walled cysts are scattered in the lung fields (case 20, lymphocytic interstitial pneumonia). 4B: In case 20, dense interstitial lymphoid infiltrates, including lymphocytes and plasma cells with type II cell hyperplasia, are observed. The alveolar septal interstitium is expanded by fibrosis. Lymphoid follicles are present. 5A: In case 24, a case of acute interstitial pneumonia, a patchy high-density shadow and bronchograms are seen in the CT image. 5B: Case 24 exhibits diffuse alveolar damage by light microscopy. The alveolar septal interstitium is expanded. Fibroblast proliferation and hyaline membrane disease are shown.

High-resolution chest CT images (HRCT) showed ground glass attenuation in 25 patients. CT scans of the two patients with PAP showed the specific paving stone sign or ‘crazy-paving sign’. Cystic air spaces were observed in four patients (NSIP, post-infectious ILD, lipoid pneumonia, and LIP), reticulation was observed in five patients (one case of EAA and four cases of NSIP), and one patient with PAM showed micronodulation throughout both lungs. Pathologic analysis showed consolidation in five cases of AIP, among which one had SLE-related chDPLD. In addition, traction bronchiectasis was observed in one patient with NSIP. No patient in our study had honeycombing or peribronchiolar thickness. Some CT images of patients were showed in Fig. 2.

### 4. Lung function test and bronchoalveolar lavage

Fourteen patients underwent a single lung-function test. For three of these patients (PAM, BOOP, and NSIP), results were normal. Lung function tests of four patients showed mixed ventilatory dysfunction (3 cases of NSIP and 1 case of LIP), and tests of the remaining seven patients showed restrictive ventilatory dysfunction.

Eight patients underwent fiberoptic bronchoscopy because of pulmonary diffuse lesion of unknown nature in CT. No obvious abnormalities were found during the procedure except for a milky appearance of the bronchoalveolar fluid in 2 patients with PAP.

### 5. Pathology findings

Among the largest group of chDPLD, none of NSIP patients had a fibrosing pattern on pathology. Five showed a cellular pattern, while four showed a mixed pattern. The remaining cases of chDPLD had their own characteristic pathology (Fig. 2).

## Discussion

Diffuse parenchymal lung diseases in children are generally thought to be rare, and different prevalence rates have been reported. In a national survey of chDPLD, the prevalence of chDPLD in different areas were 3.6 cases/million to 8.8% [4–6]. The reason for the variation in prevalence has yet to be investigated. Some evidence had been shown for the role of genetic factors in the development of chDPLD [7–9]. Yang et al [7] reported that significant transcriptional differences exist in familial and sporadic idiopathic interstitial pneumonia. However, we have not observed family cluster phenomenon in our study. Aside from genetic factors, our results may indicate that environmental factors play an important role in the development of chDPLD.. EAA can be associated with exposure to a variety of finely dispersed environmental antigens [10], occupational exposures [11], and pet birds [12]. We have three cases of exposure-related ILD, two cases of EAA and one case of lipoid pneumonia. The two cases of EAA had exposure to pet birds. The case of lipoid pneumonia had an underlining disease of gastro-esophageal reflux disease

After the detection of possible environmental factors and reviewing the patient’s history, a thorough examination of signs, symptoms, and imaging characteristics is essential. There may be clues leading to the diagnosis of chDPLD, according to Deterding R [13]. In our study, cough, dyspnea, tachypnea and retractions were the most common symptoms and signs observed. A few patients had fever, resembling bronchitis or pneumonia. However, patients with DPLP are refractory to routine antibiotic therapy, and this fact should remind pediatricians of considering chDPLD. Additionally, nail clubbing was seen in 8 patients, and this is a very important sign of chronic pulmonary diseases. Age may provide a clue to the specific type of chDPLD. In the United Kingdom and Ireland, a peak age of onset younger than one year was observed [4]. In our study, patients younger than one year were not found, and the peak age of onset was 3–7 years with a median of 6 years. All nine patients with NSIP were older than 3 years. In contrast, three of the four patients with AIP were younger than 3 years.

HRCT is a very helpful tool in the diagnosis of chDPLD, and its diagnostic and prognostic roles have been well documented. Several computer systems for image feature analyzing of DPLD were developed [14–17]. A computer-aided scheme to detect early DPLD using low-dose CT examinations was developed in 2011. This scheme yielded 80.0% sensitivity and 85.7% specificity [15]. In our study, the basic pattern of chDPLD was ground glass opacity. In some cases, specific patterns, such as a paving-stone sign, micronodulation, or thin-walled cysts may indicate certain types of chDPLD. The paving-stone sign has been found to have a high sensitivity and specificity in diagnosing PAP even without support from clinical data [18]. The diagnosis of neuroendocrine cell hyperplasia of infancy, a kind of chDPLD specific to infancy, was made on the basis of the clinical and CT findings, independent of the lung biopsy results [19]. The most common initial HRCT findings were ground-glass opacities that were in the middle lobe/ lingula. The diagnostic performance of HRCT can be increased by adding clinical data [18]. Therefore, communication between radiologists and clinicians is important. In addition, a study on idiopathic interstitial pneumonia showed that HRCT could identify complications such as infection, malignancy, acute exacerbation and pulmonary hypertension [20].

Although pathologic diagnosis is a very important way to confirm the diagnosis of chDPLD and to classify the disease into a specific category, it is not the golden standard in diagnosis of DPLD [21]. The clinical- radiologic- pathologic diagnosis pattern has been emphasized since 2002 [3]. The pathologic result should be interpreted with the clinical information. For example, in this study, we had two patients whom pathology alone would diagnose with LIP. However, one was HIV-positive. The most common DPLD in HIV-infected children is LIP [22]. Eventually, this case was classified into the category of AIDS-related chDPLD. Similarly, a patient with AIP pathologically was classified into SLE-related chDPLD. Thus, confirmation of the diagnosis requires that clinicians, radiologists and pathologists confer together.

There are limitations in our study. Firstly, the sample size was too small. Most of parents would refuse to do biopsy even when chDPLD was highly suspected. Secondly, genetic testing was not used in diagnosis. This will be included in our future studies. Thirdly, the treatment and follow-up information was not collected systematically. Such information is important for guiding the treatment of chDPLD.

In conclusion, chDPLD remains rare and complex. Lung biopsy is always a crucial approach for the final diagnosis. However, clinical and imaging features should be carefully studied for their value in indicating a specific chDPLD. Moreover, the collaboration of pediatricians, radiologists and pathologists is essential for diagnosing chDPLD.
